# Low Molecular Weight Chitosan-Coated PLGA Nanoparticles for Pulmonary Delivery of Tobramycin for Cystic Fibrosis

**DOI:** 10.3390/ph11010028

**Published:** 2018-03-08

**Authors:** Nusaiba K. Al-Nemrawi, Nid’’A H. Alshraiedeh, Aref L. Zayed, Bashar M. Altaani

**Affiliations:** 1 Department of Pharmaceutical Technology, Faculty of Pharmacy, Jordan University of Science and Technology, Irbid 22110, Jordan; nhalshraiedeh@just.edu.jo (N.H.A.); altaani@just.edu.jo (B.M.A.); 2 Department of Medicinal Chemistry and Pharmacognosy, Faculty of Pharmacy, Jordan University of Science and Technology, Irbid 22110, Jordan; alzayed@just.edu.jo

**Keywords:** mucoadhesive nanoparticle, chitosan, PLGA, tobramycin, cystic fibrosis

## Abstract

(1) Background: Poly(lactic-co-glycolic acid) (PLGA) nanoparticles (NPs) loaded with Tobramycin were prepared using a solvent-evaporation method. (2) Methods: The NPs were coated with low molecular weight chitosan (LMWC) to enhance the mucoadhesiveness of PLGA-NPs. The following *w*/*w* ratios of tobramycin to LMWC were prepared: control (0:0.50), F_0_ (1:0.25), F_0_._5_ (1:0.5), and F_1_ (1:1). (3) Results: The results showed that the size of the particles increased from 220.7 nm to 575.77 nm as the concentration of LMWC used in the formulation increased. The surface charge was also affected by the amount of LMWC, where uncoated-PLGA nanoparticles had negative charges (−2.8 mV), while coated-PLGA NPs had positive charges (+33.47 to +50.13 mV). SEM confirmed the size and the spherical homogeneous morphology of the NPs. Coating the NPs with LMWC enhanced the mucoadhesive properties of the NPs and sustained the tobramycin release over two days. Finally, all NPs had antimicrobial activity that increased as the amount of LMWC increased. (4) Conclusion: In conclusion, the formulation of mucoadhesive, controlled-release, tobramycin-LMWC-PLGA nanoparticles for the treatment of *P. aeruginosa* in cystic fibrosis patients is possible, and their properties could be controlled by controlling the concentration of LMWC.

## 1. Introduction

Cystic fibrosis (CF) is a life-threatening chronic pulmonary infection where the patient’s lungs secrete a highly viscous mucus which impairs mucociliary clearance. This mucus acts as a medium that supports bacterial infections such as *Staphylococcus aureus, Haemophilus influenzae,* and *Pseudomonas aeruginosa*. Inflammation and infection of the lungs cause injury and structural changes, including the stimulation of the release of neutrophil chemoattractants from epithelial cells and neutrophils. Further, neutrophil breakdown leads to increased viscosity of the mucus [1]. All of these conditions significantly impact the success of antibiotic treatment in CF patients [2]. In recent years, inhaled antibiotics have received greater attention, especially in the treatment of pulmonary infections related to CF. Inhaled antibiotics deliver high drug concentrations directly to the site of infection, which reduces the side effects and improves the therapeutic potential of the antibiotic against microorganisms such as *Pseudomonas aeruginosa* [3,4].

Tobramycin is one of the important antibiotics used for lung infections that develop secondary to CF and are caused by *P. aeruginosa.* Aerosolized tobramycin has been used clinically to reduce the systemic toxicity of tobramycin (nephrotoxicity and ototoxicity) and enhance its concentrations in the lungs. The new dry powder inhalers (DPIs) such as Tobramycin Inhalation Powder™ (TIP) showed similar efficacy to the nebulized inhalation solution [5,6]. These formulations have the advantages of improved patient convenience, portability, and reduced treatment time. However, these formulations face some problems such as the limited penetration of the drug through the thick mucosa of CF patients [3]. Therefore, novel strategies for improving the delivery and the deep penetration of tobramycin through the mucosa, such as the use of nanoparticles (NPs), could enhance the overall therapy outcomes [7,8].

Recently, it has been reported that polymeric NPs have the potential to penetrate mucus and overcome the steric inhibition that results from the dense mucin fiber meshes [9]. Furthermore, controlling NP surface properties such as charge and degree of lipophilicity could reduce the unfavorable chemical properties of the free molecule [10]. NPs cross the mucosal epithelium better than microspheres, since both the microfold (M) cells overlying the mucosa-associated lymphoid tissue (MALT) and the epithelial cells are involved in the transport of NPs [11,12]. Therefore, the use of nanoparticles seems very beneficial for antibiotic inhalation.

Biodegradable polymeric NPs may control the drug level at the infection site, which is expected to enhance the drug efficacy, to decrease the number of doses administered, and to reduce the side effects. NPs prepared from poly(lactic-co-glycolic acid) (PLGA) are reported to be safe to the lung and do not induce lung tissue damage. Furthermore, in vitro cytotoxicity studies have shown that PLGA has no manifest toxicity against healthy lung macrophages or CF bronchial cells [13].

PLGA NPs have been used to control the delivery of antibiotics in several ways. They have been used in the treatment of *Mycobacterium tuberculosis*, *P. aeruginosa*, *Staphylococcus aureus*, and *Escherichia coli* infections through different routes of administration [8,14,15,16]. The major challenge in using PLGA NPs is going through the thick mucin barrier and reaching the infected cells of the lung to interact with the defective environment. For that reason, other polymers have been used to modify PLGA NPs to improve their effectiveness, to enhance their deposition and their retention in the lungs, and to prevent their exhalation [3]. 

Bioadhesive polymers can improve the effectiveness of a therapy by increasing the residence time of the formulation in the lungs. Among the carbohydrates generally used in the pharmaceutical field, chitosan, a copolymer of glucosamine and *N*-acetylglucosamine, has a well-known bioadhesive nature. It establishes electrostatic interactions with the sialic groups of mucins in the mucus layer. In addition, it was demonstrated that chitosan could enhance the absorption of hydrophilic molecules by promoting a structural reorganization of the tight junction-associated proteins [17,18].

In this study, the aim was to design and to develop a pulmonary mucoadhesive nanoparticulate system for tobramycin and to demonstrate its antimicrobial efficacy. Further, the enhancement of the mucoadhesive properties of these NPs to mucin was one of the major aims. The surface properties (charge) and the bulk properties (size and entrapment efficiency) in relation to formulation variables are to be evaluated. Finally, the antimicrobial activity of the developed NPs against *P. aeruginosa* is to be investigated. 

## 2. Materials and Methods

### 2.1. Materials

Low molecular weight chitosan (90–150 kDa), poly(lactic-coglycolic acid) (PLGA) with a lactide to glycolide ratio of 50:50 (MW 40–75 kDa) and poly(vinyl alcohol) (PVA) (MW 13–23 kDa, 87–89% hydrolyzed) were purchased from Sigma-Aldrich (St. Louis, MO, USA). Tobramycin and mucin (type III) were purchased from Acros (Morris, NJ, USA). All other chemicals and reagents used were of analytical grade.

### 2.2. Preparation of PLGA and LMWC-PLGA NPs 

The NPs were prepared according to the method described by Bodmeier et al. with some modifications [19]. Briefly, 100 mg of PLGA was dissolved in 10 mL of dichloromethane (DCM) to prepare a 1% solution. In a different beaker, 80 mg of PVA (0.4% *w*/*v*) and 200 mg of tobramycin (1% *w*/*v*) were dissolved in 20 mL of deionized water. The organic phase was then dropped into the aqueous phase under sonication for 3 min (Amplitude 40%, pulse on 30 s, pulse off 5 s) using an ultra-sonic processor (Sonic, Vibra cell, SON-1 VCX130, probe number 422-17, Newtown, CT, USA). The formed oil in water (o/w) emulsion was mixed with 20 mL of 0.5% PVA and stirred using a magnetic stirrer to remove excess DCM for 2 h. The nanoparticles were separated by centrifugation (Thermo scientific, Darmstadt, Germany) at 10,000 rpm for 30 min. Then, the NPs were washed three times with deionized water. Finally, the sample was freeze-dried (Telstar, Spain) for 48 h at −80 °C to obtain the NPs [20]. In the case of the formulation of LMWC-PLGA NPs, the same procedure that was described previously was followed, but specific amounts of LMWC were added to the aqueous phase. By the end, five formulations were prepared and studied. One of these formulations did not contain chitosan, but was loaded with tobramycin and was called F_0_ [21]. Another three formulations were prepared using 100 mg of PLGA (1%), which was dissolved in 10 mL of DCM and 80 mg of PVA (0.4% *w*/*v*); 200 mg of tobramycin (1% *w*/*v*); and varying concentrations of LMWC, which were then dissolved in 20 mL of deionized water. Thus, F_0.25_ contained 0.25% *w*/*v* of LMWC in the aqueous phase, F_0.5_ contained 0.5% *w*/*v* of LMWC in the aqueous phase and F_1_ contained 1% *w*/*v* of LMWC in the aqueous phase. Finally, one more formula was prepared using 0.5% *w*/*v* of LMWC in the aqueous phase, but no tobramycin was added, and this was called the Control. The compositions of all the formulations are given in Table 1. 

### 2.3. Characterization of PLGA and LMWC-PLGA Nanoparticles

The mean particle size (PS), the polydispersity index (PDI) and the zeta potential (ZP) of NPs were determined using a Zetasizer nano ZS90 instrument (Malvern Instruments, Malvern, UK) at 25 °C using dynamic light scattering (DLS). All measurements were carried out in triplicate (*n* = 3). The zeta potentials were determined by placing diluted samples of the NPs in deionized water at 25 °C in clear disposable zeta cells. Based on the Smoluchowski equation, the electrophoretic mobility between the electrodes was converted to a zeta potential. All measurements were carried out in triplicate (*n* = 3).

The morphologies of PLGA and LMWC-PLGA NPs were explored using Scanning Electron Microscopy (SEM) (Thermo scientific, Darmstadt, Germany). Furthermore, the effect of the surface modification caused by LMWC was investigated using SEM. The samples were coated with carbon film prior to their analysis and were then studied under a microscope. 

The Fourier-transform infrared (FT-IR) spectra of PLGA and LMWC-PLGA NPs were compared to study the interaction between PLGA and LMWC. A Shimadzu IR spectrophotometer (Shimadzu, Kyoto, Japan) with a high-performance diamond single-bounce ATR accessory (wave number 400–4000 cm^−1^, resolution 4 cm^−1^ with 64 scans per spectrum) was used to record the results. 

### 2.4. Drug Entrapment Efficiency and Loading Capacity

The drug entrapment efficiency (*EE*) and loading capacity (*LC*) were determined. The *EE* was determined by finding out the free amount of tobramycin that was not encapsulated in the NPs in relation to the total amount of tobramycin used in each formulation. During the formulation of the NPs, the resulting supernatant from centrifugation was analyzed for free tobramycin using the HPLC-UV method [22]. The encapsulated amount of tobramycin was calculated by subtracting the free amount of tobramycin from the total amount in the dispersion (*n* = 3). The *EE* was calculated according to the following equations:

$$EE=\;\frac{\left(\mathrm{Total}\;\mathrm{amount}\;\mathrm{of}\;\mathrm{tobramycin}\;-\;\mathrm{free}\;\mathrm{amount}\;\mathrm{of}\;\mathrm{tobramycin}\right)\times 100\%}{\mathrm{Total}\;\mathrm{amount}\;\mathrm{of}\;\mathrm{tobramycin}}$$

The *LC* was determined by dividing the amount of tobramycin trapped in the nanoparticles by the total sample weight as follows:

$$LC=\;\frac{Tobramycin\;in\;NPs\;\times \;100\%}{NPs\;weight}$$

Tobramycin was measured using HPLC-UV (Shimadzu, Japan) according to Russ et al. with some modifications. The C_18_ column (5 µm, 4.6 × 250 mm) was used at 25 °C and the λ_max_ was set at 365 nm. The mobile phase was prepared by dissolving 2.0 g of tris(hydroxymethy1) aminomethane in 800 mL of water, and then, 20 mL of 1 N sulfuric acid was added. Then, the solution was added to 1200 mL of acetonitrile. The flow rate was 1.0 mL/min. The samples were derivatized as follows: 400 μL of each sample was added to 1 mL of 2,4-Dinitroflurobenzene reagent (10 mg/mL alcohol) and 1 mL of Tris (hydroxymethyl) aminomethane reagent (15 mg/mL in water/dimethylsulfoxide, 20/80; *v*/*v*) in a 5.0 mL volumetric flask. The flasks were shaken, covered and put in an oven at 60 ± 2 °C for 50 min. After that, the flasks were removed and allowed to stand for 10 min at room temperature. Then, the samples were diluted with acetonitrile up to 5.0 mL. 

### 2.5. Investigation of the Mucoadhesive Properties of PLGA and LMWC-PLGA Nanoparticles

The mucoadhesive properties of LMWC-PLGA NPs were evaluated by measuring the changes in the zeta potential (ZP) of the NPs when interacting with negatively charged mucin. A mucin stock suspension was prepared by adding mucin powder type III to a Tris buffer (pH 6.8) at a concentration of 1% *w*/*w*. 

The mucin suspension was stirred overnight at 37 °C; then, it was homogenized by ultrasonication at 40% amplitude for 3 min, and centrifuged at 4000 rpm for 20 min. After that, the NPs were incubated at 37 °C with the mucin suspension. The ZP was measured at the beginning of the experiment and after 1, 2, 3, and 4 h of incubation. The alteration of the ZP of the NPs was used as an indicator that the NPs had interacted with the mucin [23,24]. The ZP was measured as explained in Section 2.3. 

### 2.6. In Vitro Drug Release

To determine the in vitro release of the drug, tobramycin-loaded nanoparticles were dispersed into 2 mL of a phosphate buffer solution with a pH of 7.4. Then, the suspension was put into a cellulose dialysis bag (molecular weight cutoff 12–14 KDa) (Spectra por, Rancho Dominguez, CA, USA). The dialysis bags were soaked into tubes that contained 8 mL of the phosphate buffer solution as a dissolution medium. After that, the tubes were transferred into a 37.0 °C water bath that shook at 100 rpm [25]. At allocated time intervals, 5 mL of dialysis solution was withdrawn, and this volume was replaced by fresh dissolution media. The tobramycin concentration in each sample was determined by the same HPLC-UV procedure used for the determination of the *EE*. 

### 2.7. Antimicrobial Activity of Tobramycin Nanoparticles

The Minimal Inhibitory Concentration (MIC) was measured using *P. aeruginosa* (PA01). *P. aeruginosa* was grown in LB overnight at 37 °C with an agitation rate of 100 min^−1^. Then, it was diluted to an optical density (OD_550_) equivalent to 1 × 10^7^ cfu/mL. Aliquots of 100 μL of OD_550_, overnight-adjusted culture, were added in triplicate to each well of a 96-well microtiter plate. Each plate contained 100 μL of varying concentrations of tobramycin (free tobramycin or the equivalent amount of tobramycin in F_0_, F_0.25_, F_0.5_ and F_1_). The weight of the nanoparticles used in the preparation of these varying concentrations was determined depending on the *EE* in each formula. The concentrations were prepared using the serial dilution technique; then, the plates were incubated for 24 h at 37 °C in an orbital incubator (JSR Shaking incubator, Gongju, Korea). A negative control which consisted of uninoculated broth was also included in triplicate on each plate. The MIC was determined as the lowest concentration for which no growth was visually observed in the inoculated wells. 

### 2.8. Preparation of Biofilms Using the Calgary Biofilm Device/MBEC Assay

The biofilm of the *Pseudomonas* bacteria was grown using the Calgary Biofilm Device (commercially available as the MBEC Assay™ for Physiology & Genetics (P & G) (Innovotech Inc., Edmonton, AB, Canada)) according to the previously described method by Ceri et al. [26]. This device consists of a 96-well plate with a lid bearing polycarbonate (PC) pegs, which protrude into each well containing bacterial culture. This allows the growth of 96 identical biofilms per device. An overnight culture of bacteria was adjusted to an optical density (OD_550_) equivalent to 1 × 10^7^ cfu/mL. Each well of the Calgary Biofilm Device was inoculated with an aliquot of 150 μL of the standardized bacterial suspension. The lid containing the pegs was placed carefully into the wells and the CBD was incubated at 37 °C within an orbital incubator for 48 h in a humidified compartment to allow the formation of biofilms. After the first 24 h of incubation, the bacterial inoculum was replaced by a fresh growth medium. Following 48 h of incubation, pegs were placed in a fresh 96-well rinse plate (each well containing 200 μL of fresh growth medium) and were gently rinsed to remove any planktonic or loosely attached bacteria before their exposure to tobramycin F_0_, F_0.25_, F_0.5_ and F_1_. 

The minimum biofilm eradication concentrations (MBECs) were determined in triplicate [26]. Briefly, the 48-h old biofilm was challenged with a range of concentrations of tobramycin—F_0_, F_0.25_, F_0.5_ and F_1_—for 24 h at 37 °C in a gyrorotary incubator (Binder, Tuttlingen, Germany). After 24 h of this challenge, the pegs were gently rinsed three times in phosphate-buffered saline (PBS); then, they were placed in a second 96-well plate (recovery plate) containing 200 μL of fresh growth media and were sonicated for 10 min. Following sonication, the lid carrying the pegs was discarded and the recovery plates were incubated for 24 h at 37 °C in the orbital incubator. The MBEC was determined as the lowest antibiotic concentration that prevented the regrowth of the bacteria from the treated biofilm. A negative control was also included.

## 3. Results

PLGA nanoparticles were prepared using the emulsion-solvent evaporation method. The surfaces of the NPs were modified with LMWC in order to enhance their mucoadhesive properties. 

### 3.1. Characterization of PLGA and LMWC-PLGA Nanoparticles

**Figure 1:**
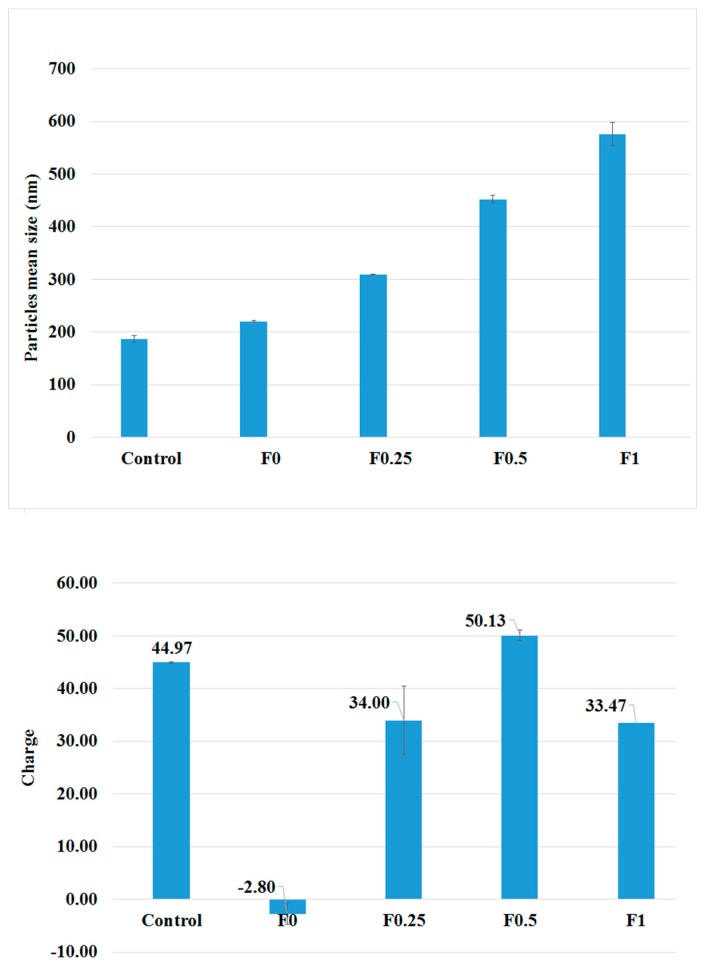
The particles’ mean sizes (**top**) and the surface charge profiles (**down**) of PLGA and LMWC-PLGA NPs with different concentrations of LMWC.

The mean particle size and the zeta potential of the NPs that were prepared with and without LMWC are introduced in Figure 1. The PLGA nanoparticles that were prepared without tobramycin (control) had the smallest size among all the NPs under study (187 ± 6.19 nm). When tobramycin was loaded, the sizes of the particles increased. Further, coating the PLGA NPs with LMWC showed a direct relation to the particle size, and the increment in size was related to the concentration of LMWC. Nanoparticles that were prepared using 50, 100, and 200 mg of LMWC resulted in particles with average sizes of 309.57 ± 1.12, 451.8 ± 7.19, and 575.77 ± 2.67 nm, respectively, whereas the uncoated NPs had a size of 220.7 ± 1.77 nm. 

**Figure 2:**
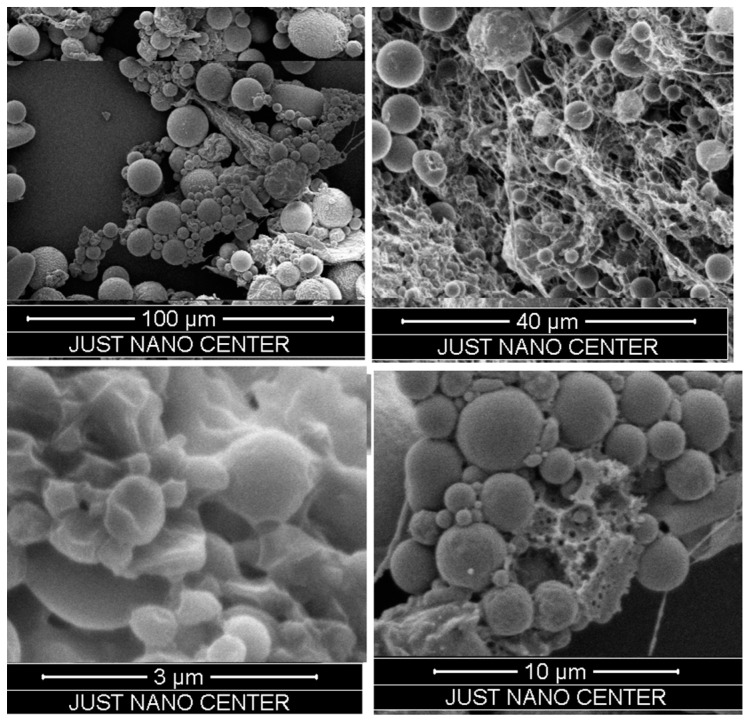
Scanning electron microscope images of LMWC-PLGA nanoparticles showed nanosized spherical particles.

The mean diameter that was measured by a Zetasizer analysis of the NPs was confirmed by SEM. The nanoparticles showed a spherical morphology, as shown in Figure 2. Coating the NPs with LMWC affected the surface charge of the NPs. The PLGA nanoparticles had a negative zeta potential (−2.8 ± 0.1 mV) while the LMWC-PLGA nanoparticles had positive charges. Nanoparticles that were prepared using 50, 100, and 200 mg of LMWC gave particles with average charges of +34.0 ± 1.9, +50.1 ± 6.5, and +33.47 ± 1.0 mV respectively. The surface charge was found to have no relation to the concentration of LMWC, as shown in Table 2. 

**Figure 3:**
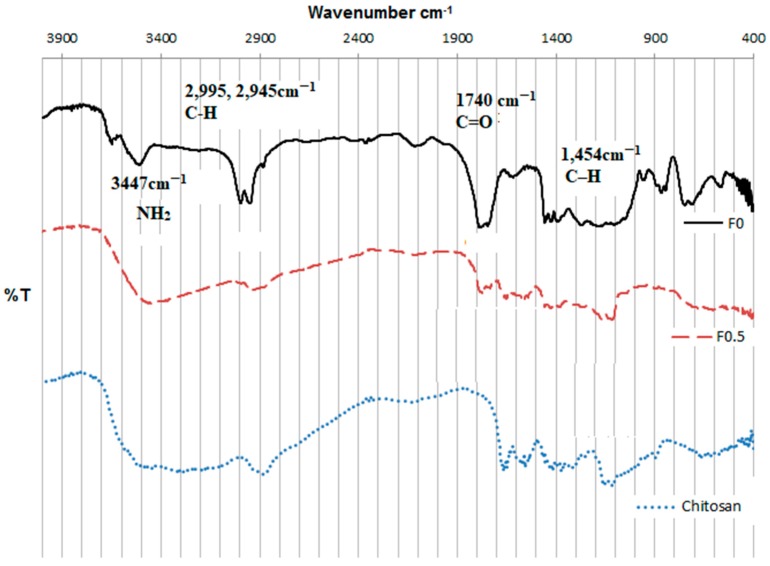
FT-IR spectra of the LMWC raw material, the PLGA NPs (F_0_), and the LMWC-PLGA NPs (F_0.5_).

Although the changes in the surface charges when the NPs were coated with LMWC provided proof of a successful coating, the coating was further proved using FT-IR studies. The IR spectra of the F_0_ and F_0.5_ nanoparticles are shown in Figure 3. 

Figure 3 compares the FTIR spectra of the uncoated PLGA NPs (F_0_) and the LMWC-PLGA NPs (F_0.5_) in reference to the chitosan spectrum. A characteristic band was observed at 3447 cm^−1^ related to the –NH_2_ and –OH groups stretching in the LMWC (Figure 3). A band corresponding to amine stretching at 1110 cm^−1^ was also seen in the infrared spectrum of the native LMWC (Figure 3). The characteristic peaks of LMWC that were observed in F_0.5_ but not F_0_ clearly proved that LMWCs were deposited on the surface of PLGA nanoparticles during the coating process. From the PLGA NPs (F_0_ in Figure 3), the C–H stretching in the methyl groups at 1454 cm^−1^, the C=O at 1740 cm^−1^, the C–H stretching vibrations at 2995 and 2945 cm^−1^, and the OH stretching at approximately 3500 cm^−1^ can be noticed. The C=O peak frequency was also noticed in the spectrum of the LMWC-coated, PLGA nanoparticles (F_0.5_ in Figure 3), which resulted from the PLGA core of the coated nanoparticles. These results are in agreement with other studies conducted with similar nanoparticles [27,28]. 

### 3.2. Drug Entrapment Efficiency

In this study, the *EE*% of tobramycin in the NPs was very high. The *EE* of tobramycin in the NPs ranged from 83.74% (167.48 mg) to 88.47% (176.94 mg), as shown in Table 2. The coating with LMWC did not show any obvious effect on the *EE* of tobramycin. 

### 3.3. Mucoadhesive Properties of PLGA and LMWC-PLGA Nanoparticles

**Figure 4:**
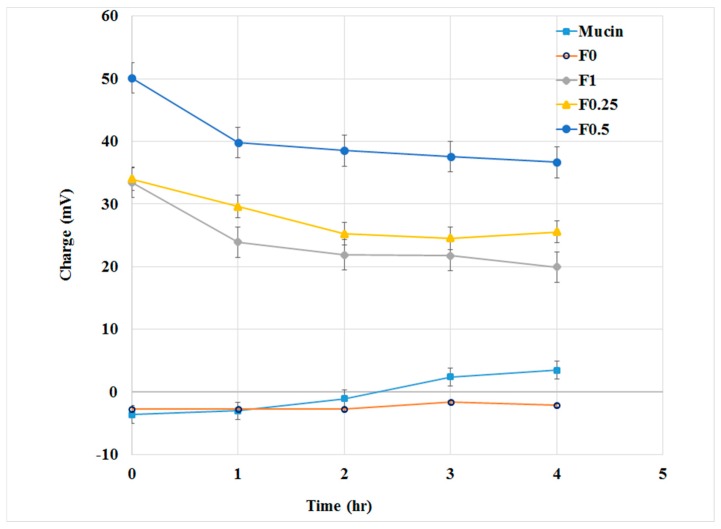
Assessment of the zeta potential (ZP) of the PLGA and LMWC-PLGA NPs throughout incubation in the aqueous dispersion of mucin.

In order to evaluate the interaction between LMWC-PLGA NPs and mucin, zeta potential measurements of the mucin-NPs’ dispersions were conducted, and the results are presented in Figure 4. At the beginning of the experiment, PLGA nanoparticles showed negative zeta potentials while all LMWC-PLGA NPs had positive zeta potentials. 

As the incubation time passed, the potentials of the LMWC-PLGA NPs decreased slightly due to their interaction with mucin, but still recorded high positive values in comparison to PLGA NPs. 

### 3.4. In Vitro Drug Release

**Figure 5:**
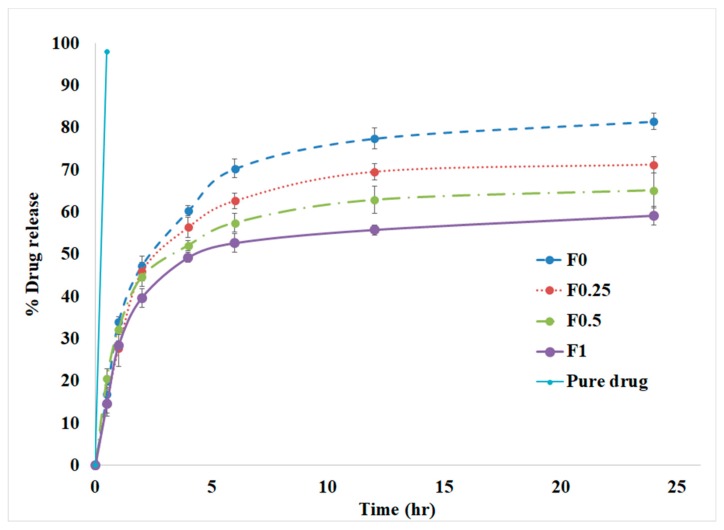
Cumulative release profiles of tobramycin-loaded PLGA and LMWC-PLGA NPs in phosphate-buffered saline (pH = 7.4) at 37 °C.

The release of tobramycin in vitro from different nanoparticle formulations is shown in Figure 5. The pure drug was completely available in the solution (99%) after 30 min. It is evident that the component of the NPs affected the release of tobramycin in vitro. All NPs showed the emergence of an initial burst of release before the first 2 h, followed by a relatively slow release rate of the drug. The release of tobramycin from the coated NPs was slower in comparison to the uncoated NPs. After two days, the uncoated PLGA nanoparticles released 86.82 ± 2.3% of the entrapped drug, while the LMWC-PLLGA NPs released 71.81 ± 3.1, 65.52 ± 1.8, and 59.53 ± 2.0% for F_0.25_, F_0.5_ and F_1_, respectively.

### 3.5. Antimicrobial Activity of Tobramycin Nanoparticles

The antimicrobial activity of tobramycin as a raw material or when it was loaded in the different NPs prepared in this study was tested against a planktonic culture of *P. aeruginosa* (PA01). The MIC value for each formulation was measured. The results showed that tobramycin alone and all the four formulas (F_0_, F_0.25_, F_0.5_, and F_1_) inhibited bacterial growth, whereas the control NPs (not loaded with tobramycin) did not show any bacterial inhibition. Tobramycin alone had an MIC value of 1 μg/mL, while F_0_, F_0.25_, F_0.5_, and F_1_ had MIC values of 128.15, 32.25, 4.95, and 2.9, respectively. The results are summarized in Table 3. 

### 3.6. Effect of Tobramycin PLGA NPs on P. aeruginosa Biofilms

The *P. aeruginosa* (PA01) biofilms grown on the Calgary Biofilm device were challenged with tobramycin, F_0_, F_0.25_, F_0.5_, and F_1_. As shown in Table 3, tobramycin had an MBEC value of 7.8 compared to 512, 250, 15.6, and 125 for F_0_, F_0.25_, F_0.5_, and F_1_, respectively.

## 4. Discussion

The presence or absence of LMWC in the formulation and its concentration affected the PLGA NPs’ sizes and surface charges. Chitosan is a hydrophilic polymer that swells when it is dispersed in water, and the water viscosity increases as the chitosan concentration increases [29,30]. The greater increase in particle sizes when the NPs were coated with LMWC was maybe related to its effect on the viscosity of the adjacent liquid layer next to the NPs. As the LMWC concentration increases, this layer is expected to enlarge and become more viscous. This fact may explain the increment in the size of the surface-modified PLGA particles. Another reason that may explain this increment in size is the larger amount of LMWC that was deposited on the surface of the PLGA nanoparticles as the concentration of LMWC increased [23,31].

The PLGA nanoparticles had a negative zeta potential because of the PLGA free carboxyl groups on the surface of the NPs. On the other hand, the LMWC-PLGA nanoparticles had high positive charges due to the new amine functional groups on the NPs’ surfaces that is related to LMWC [24]. Although the size of the NPs increased as the amount of LMWC used in the formulation increased, the zeta potential did not show the same direct relation to the amount of LMWC. It has been shown that the charge density on the surface of chitosan nanoparticles depends on the nanoparticle size and the amount of chitosan that is used in the preparation [32,33]. It is expected that as the amount of chitosan used in the preparation of the NPs increases, the number of free amine groups will also increase, which will lead to higher zeta potentials. On the other hand, the effect of the particle size on the zeta potential is more complicated. In general, the smaller the particles of any given sample, the greater the total surface area per weight, but at the same time, the individual particles themselves have smaller surface areas [34]. Therefore, it was observed that as the sizes of the chitosan NPs increased, the zeta potentials increased until a maximum value was reached, after which the charge began to decrease again. Finally, the high positive surface charge of LMWC-PLGA NPs is expected to prevent the aggregation of the particles [30,35]. 

Electrostatic interaction was suggested as the mechanism that explained the mucoadhesive interaction of the LMWC with the mucin. The zeta potential decreased after the incubation with mucin in all LMWC-coated nanoparticles. The interaction between the sialic groups of the mucin (negatively charged) with the surface layer of the LMWC (positively charged) on the LMWC-PLGA NPs was expected to decrease the zeta potential. After four hours of incubation, the surface charges of the LMWC-PLGA NPs were almost the same, which may indicate the stability of the electrostatic interaction between the LMWC-coated nanoparticles and the mucin. On the other hand, the uncoated PLGA NPs (F_0_) showed a stable negative charge, which indicates that there was no interaction with the mucin [36,37].

All the nanoparticle formulations showed a biphasic release, with an initial burst release of the drug, followed by a sustained release. This biphasic behavior of the drug release was mentioned in other works related to PLGA NPs. The high burst release at the beginning was related to the free drug or weakly bonded drug in the nanoparticles [38,39,40]. It is well-known that PLGA NPs degrade through the hydrolysis of the ester linkages between their lactic and glycolic acid oligomeres in an aqueous medium, which then causes the drug to be released [38]. On the other hand, LMWC swells in water and forms a hydrogel layer that controls drug diffusion. Coating the NPs with LMWC slowed down the drug release, and this reduction was related to the amount of LMWC used in the formulation. This indicates that the LMWC layer on the nanoparticle surface serves as an additional barrier against drug diffusion [10]. 

All the formulas in this study were found to inhibit the bacterial growth except the control formula, which was not loaded with any drug. There was a clear difference in the MIC between the four different formulations, where F_1_ had the lowest MIC with a value of 2.9, and F_0_ had the highest MIC with a value of 128.15. The MIC values may be related to different variables such as the particle’s size or the charge. The fact that the control NPs did not show any antimicrobial activity confirms that the antimicrobial activity was elicited by tobramycin and not by any other ingredient in the formulation. Although the control NPs did not show any antimicrobial activity, there was a clear relationship between the amount of the LMWC used in the formulation and the NPs’ antimicrobial activity. In general, increasing the amount of LMWC in the formulations enhanced the NPs’ microbial inhibition. This relation may be related to the ability of coated NPs to adhere well to the microbes’ membrane in comparison to the uncoated NPs, which gives the released tobramycin the opportunity to affect the microbes faster and in higher concentrations. 

Tobramycin alone exhibited the lowest MBEC, and the closest MBEC value was achieved by F_0.5_, which was double the value of tobramycin alone. Generally, the encapsulation of tobramycin into nanoparticles results in higher MBEC values, which may be a reason for the gradual release effect of the NPs, as proven by the drug release study [41,42]. The ability of the NPs to control the drug release over a long time is expected to improve the overall efficacy of the formulation in comparison to tobramycin alone [43,44].

## 5. Conclusions

A modified, mucoadhesive, tobramycin nanoparticle targeting *P. aeruginosa* for the treatment of cystic fibrosis was prepared successfully in this work. The prepared formulations could improve patient compliance due to their prolonged action, which would be beneficial in reducing the overall frequency of dosing and minimizing the side effects. Coating the NPs with LMWC enhanced the NPs’ mucoadhesion and sustained the drug release from the NPs. The concentration of chitosan that is used in the formulation is important in determining the physicochemical properties, the release and the antimicrobial activity of the formulation. 

## Figures and Tables

**Table 1:** The composition of different nanoparticle (NP) formulations in each formula (mg).

Component	Formulation				
	Control	F_0_	F_0.25_	F_0.5_	F_1_
Tobramycin	-	200	200	200	200
Low Molecular Weight Chitosan (LMWC)	100	-	50	100	200
Poly(vinyl alcohol) (PVA)	80	80	80	80	80
Poly(lactic-co-glycolic acid) (PLGA) 50:50	100	100	100	100	100

**Table 2:** Effects of different LMWC concentrations in the aqueous phase during the creation of PLGA NPs loaded with tobramycin based on size (nm), polydispersity index (PDI), charge (mV), drug entrapment efficiency (*EE*) (percentage), and loading capacity (*LC*) (percentage).

Formulae	Size (nm)	PDI	Zeta Potential (mV)	*EE* (%)	*LC* (%)
Control	187.00 ± 6.19	0.206 ±0.014	+45.00 ± 4.30	NA	NA
F_0_	220.70 ± 1.77	0.194 ±0.002	−2.80 ± 0.10	85.34%	45.92%
F_0.25_	309.57 ± 1.12	0.295 ±0.011	+34.00 ± 1.90	88.47%	43.15%
F_0.5_	451.80 ± 7.19	0.297 ±0.106	+50.13 ± 6.50	87.20%	37.33%
F_1_	575.77 ± 2.67	0.319 ±0.130	+33.47 ± 1.00	83.74%	30.87%

**Table 3:** The antimicrobial effect (MIC) (μg/mL) and the minimum biofilm eradication concentration (MBEC) (μg/mL) of tobramycin, PLGA, and LMWC-PLGA NPs.

Formula	MIC (μg/mL)	MBEC (μg/mL)
Tobramycin	1	7.8
F_0_	128.15	512
F_0.25_	32.25	250
F_0.5_	4.95	15.6
F_1_	2.9	125
